# Minority Stress, Resilience, and Trouble Falling Asleep Among Gender and Sexual Minority Adolescents

**DOI:** 10.1002/jad.12520

**Published:** 2025-06-04

**Authors:** Will J. Beischel, René‐Marc Lavigne, Alexa Martin‐Storey, Jean‐Pascal Lemelin, Ryan J. Watson

**Affiliations:** ^1^ Department of Psychology Loyola University Chicago Chicago Illinois USA; ^2^ Département de Psychoéducation Le Groupe de recherche et d'intervention sur les adaptations sociales de l'enfance Université de Sherbrooke Longueuil Quebec Canada; ^3^ Département de Psychoéducation Le Groupe de recherche et d'intervention sur les adaptations sociales de l'enfance Université de Sherbrooke Sherbrooke Quebec Canada; ^4^ Department of Human Development and Family Sciences University of Connecticut Storrs Connecticut USA

**Keywords:** LGBTQ, minority stress, resilience, sleep quality, social safety, victimization

## Abstract

**Introduction:**

Gender and sexual minority adolescents experience greater stress and report worse sleep outcomes compared to their cisgender and/or heterosexual peers. Understanding how minority stress and resilience factors are linked to sleep health provides levers for improving sleep within these populations.

**Methods:**

Using a cross‐sectional survey of LGBTQ+ adolescents in the US conducted in 2017 (*n* = 11,282; *M*_age_ = 15.6 years; 65% white), we compared gender/sex and sexuality subgroups’ trouble falling asleep and conducted linear regressions relating trouble falling asleep to minority stress (i.e., violent victimization, bias‐based victimization, and family rejection) and resilience (i.e., familial warmth, family acceptance, gender‐affirming environments, teacher support, trusted adult at school, and presence of a gender‐sexuality alliance [GSA]) factors for both gender and sexual minority adolescents.

**Results:**

We found small but significant differences in sleep across gender/sex categories, with gender minorities and youth assigned female at birth having worse sleep than cisgender sexual minorities and youth assigned male at birth, respectively. Further, violent LGBTQ+ victimization and gender expression‐based victimization were associated with more trouble falling asleep, and familial warmth was associated with less trouble falling asleep for both groups. For cisgender sexual minorities, family rejection and gender‐based victimization were also linked with worse sleep while presence of a GSA and a trusted adult at school were linked with better sleep. For gender minorities, gender‐segregated restroom use was also linked with better sleep.

**Conclusions:**

Victimization prevention, increased access to school supports, and improved family connectedness may help enhance LGBTQ+ youth sleep quality and overall health.

Poor sleep quality, which can include trouble falling asleep and trouble staying asleep, is consistently related to a wide variety of physical and mental health issues (Biddle et al. 2019; Cappuccio et al. 2011). Though sleep varies across the lifespan, adolescence is a period in which sleep is especially negatively affected through both physiological (e.g., puberty) and psychosocial (e.g., greater educational demands) factors (Carskadon 2011). These disruptions in sleep impact many physiological, emotional, and behavioral processes that reduce adolescents’ resources for managing increasingly complex social dynamics (Goldstein and Franzen 2022; O'Leary et al. 2017; Silvers 2022). This is likely one of the reasons why poorer sleep quality is linked with a variety of negative mental health outcomes and physical health‐risk behaviors for adolescents, including increased suicidality, depression, substance use, and sedentariness (Goldstein and Franzen 2022; McKnight‐Eily et al. 2011; Owens et al. 2014; Paruthi et al. 2016). These findings support sleep quality as an important factor in developing adolescents’ emotional, mental, and physical well‐being, and highlight the role that their social environments play in this link.

Research increasingly suggests that sleep health difficulties are not equally distributed across the adolescent population. Gender/sex is one salient variable related to sleep health disparities, with women and/or gender minorities (e.g., transgender and/or nonbinary people) often having worse sleep outcomes than men and/or cisgender people (Aernout et al. 2021; Hershner et al. 2021; Levenson et al. 2021a). Sexual identity is another salient variable, with sexual minorities (e.g., gay, queer, bisexual people) often having worse sleep outcomes than their heterosexual peers (Dai et al. 2020; Li et al. 2017). However, existing studies do not assess the links between minoritized gender and sexual identities and poor sleep. From minority stress and social safety perspectives (V. R. Brooks 1981; L. M. Diamond and Alley 2022; Kelleher 2009; Meyer 2013), we sought to understand the association between trouble falling asleep and minority stress/resilience with a large sample of gender and sexual minority adolescents, as well as group differences across these identities.

In this article, we use the term “minorities” to refer to people who are minoritized on the basis of their genders and/or sexualities through systems of cisnormativity, heteronormativity, binarism, etc. Thus, “minority” does not refer necessarily to a statistical minority but rather being subject to marginalization within a social hierarchy (van Anders 2015). Therefore, the term “gender minorities” includes people who are transgender (trans), nonbinary, and/or gender questioning. The term “sexual minorities” includes people with identities including but not limited to pansexual, bisexual, queer, lesbian, and/or gay. These categories are overlapping in that some people are both gender and sexual minorities. We find these umbrella terms useful as specific identity terms (e.g., transgender) may or may not be adopted by participants, such that using these terms would be inaccurate. At times, we also use “gender/sex” to refer to the complex entanglement of gender (i.e., sociocultural features) and sex (i.e., anatomical/physiological features) without making claims as to whether a phenomenon is more related to gender, sex, or both (van Anders 2015; van Anders and Dunn 2009). For example, sleep seems to be impacted both by sex‐related physiology and gender‐related psychosocial experiences (Clancy et al. 2020; Jose and Brown 2008; Krishnan and Collop 2006).

## 1 Sleep Among Gender and Sexual Minorities

Research generally finds that gender and sexual minority adults and youth have worse sleep outcomes than cisgender heterosexual people, with differences being greatest during adolescence and young adulthood (Butler et al. 2020). Within LGBTQ+ communities, a few studies suggest that gender minority adults have pronounced sleep issues, including short sleep times, insomnia, and poor subjective sleep quality (Auer et al. 2017; Hershner et al. 2021), even when compared to cisgender sexual minority groups (Butler et al. 2020). Samples in this body of research are not often large enough to assess group differences within gender minorities or find no differences (Auer et al. 2017). However, one study found that nonbinary trans adults had the worst sleep outcomes compared to other gender and sexual minority groups (Dai and Hao 2019). There is also variation in sleep quality across sexualities, with plurisexual young adults (who report sexual attractions and/or behavior with people of multiple genders) having worse sleep outcomes than monosexual individuals (who report sexual attractions/behavior with people of one gender; Fricke and Sironi 2017).

Considering these sleep disparities in adults and the sleep changes among adolescents in general, gender and sexual minority adolescents may be especially prone to poor sleep. Indeed, Levenson and colleagues (Levenson et al. 2021a) found that gender minority adolescents reported poorer subjective sleep quality than their cisgender heterosexual peers. Similarly, sexual minority adolescents are at greater risk of having sleep problems than heterosexual adolescents (Dai et al. 2020; Nagata et al. 2023). These sleep disparities follow a more general trend of health disparities between gender and sexual minority youth and their cisgender heterosexual peers, including being at greater risk for anxiety, depression, suicide, substance misuse, HIV, and poorer physical health in general (Delozier et al. 2020; Hafeez et al. 2017; Lucassen et al. 2017). Despite this extensive work on health disparities, little work has investigated the reasons for sleep disparities in particular.

Understanding the factors linked to sleep quality among gender and sexual minority youth may be particularly important given the growing literature suggesting that sleep quality may be one underappreciated contributing factor to broader health disparities across gender/sex and sexuality. Research links insomnia symptoms (e.g., trouble falling asleep) to quality of life among trans boys (Bowen et al. 2021), and both sleep duration and sleep quality partially mediate the association between gender and sexual minoritized identities and suicidality (Clark et al. 2023; Huang et al. 2018). Longitudinal work with gender and sexual minority adolescents has further demonstrated that sleep duration and quality are prospectively linked with socioemotional outcomes such as self‐esteem and parental relationships, especially for gender minority youth (Wong et al. 2024). However, samples of gender and sexual minority youth are often not large enough to more fully understand what may be contributing to poor sleep quality or differences between gender/sex groups (e.g., binary and nonbinary trans youth) or sexual identity groups (e.g., plurisexual and monosexual youth), nor do large‐scale samples measure minority stress or resilience factors.

## 2 Minority Stress, Resilience, Social Safety, and Sleep

One established framework for understanding health disparities among minoritized groups is the minority stress model (V. R. Brooks 1981; Meyer 2003). This model posits that chronic stressors related to living in a cisnormative and heteronormative society (i.e., one that privileges and centers people who are cisgender and heterosexual) have a cumulative negative effect on minoritized people's well‐being. It also posits that minoritized people can be protected from the deleterious effects of adversity through personal (e.g., identity‐related pride) and community (e.g., social gender affirmation) resilience factors.

A growing body of work has linked sleep health, and especially subjective sleep quality (e.g., trouble falling asleep), with minority stress among gender and sexual minority people (Martin‐Storey et al. 2020). Stressors related to sleep issues in LGBTQ+ adults include internal factors (e.g., internalized transphobia), interpersonal factors (e.g., discrimination), and structural factors (e.g., lack of support for same‐sex marriage) (Martin‐Storey et al. 2020). Fewer sleep studies with LGBTQ+ samples have included resilience factors, but some work has found that community connectedness and family support are related to fewer sleep disturbances for trans adults (Eom et al. 2022; Kolp et al. 2020).

Recent theoretical work has posited that social safety (i.e., reliable social connection and protection) is a strong predictor of health and is often threatened for gender and sexual minorities, making minority stressors related to social safety such as violent victimization or parental rejection particularly good candidates for explaining health disparities (L. M. Diamond and Alley 2022). Accordingly, experiences that cue a loss of social safety may be especially pertinent for understanding sleep health. Conversely, experiences that foster feelings of belonging can calm the nervous system and may promote better sleep health. For example, adolescents who perceive their neighborhoods and schools as safe have better sleep outcomes compared to those who perceive these spaces as unsafe (Meldrum et al. 2018).

Despite research on minority stress and sleep with adults, the association between minority stress/resilience, social safety, and sleep among gender and sexual minority adolescents remains relatively unknown. Considering that adolescence is a time in which social experiences of rejection and belonging are especially salient predictors of well‐being (Arslan 2021; Tomova et al. 2021), it is crucial to understand how these experiences are linked with sleep among this population. In the minority stress literature, several variables have emerged that both represent serious threats to social safety and are linked to negative health outcomes. For one, gender and sexual minority youth can be the victims of violent victimization related to their minoritized status, which is associated with greater alcohol use, depression, and suicidal ideation (Baams et al. 2015; Watson et al. 2021). Second, bias‐based victimization (i.e., being treated badly because of various stigmatized identities) is associated with greater disordered eating and lower perceived school safety among gender and sexual minority youth (Lawrence et al. 2024; Lessard et al. 2020). Third, rejection from one's family because of LGBTQ+ status is associated with increased odds of cigarette smoking, suicide attempts, and substance misuse (Gamarel et al. 2020; Klein and Golub 2016). While each of these stressors are linked to negative health outcomes among LGBTQ+ youth, links with sleep quality have yet to be shown.

Though fewer studies have focused on resilience factors, existing literature provides several variables theoretically linked to social safety that may also be important for understanding sleep health among gender and sexual minority youth. Specifically, we focused on family life and school environments as two of the most important contexts in adolescents’ lives. Families vary both in how interconnected the members are in general (i.e., familial warmth) and how supported youth feel in their sexual or gender minority identity (i.e., LGBTQ+ family acceptance). These variables are linked to health‐related outcomes such as decreased depressive symptoms and increased self‐esteem (Meanley et al. 2021; Miller et al. 2020). Schools also vary in whether there are teachers supportive of LGBTQ+ people, whether students have an adult they trust at school, whether the school has a gay‐straight/gender‐sexuality alliance (GSA), and how gender affirming they are, which can include correct name and pronoun use as well as access to the correct gender‐segregated bathrooms. These school resilience factors are linked to better outcomes among gender and sexual minority youth, including higher self‐esteem, lower depressive symptoms, lower disordered eating, and lower alcohol use (Dessel et al. 2017a; Olson et al. 2016; Toomey et al. 2011; Watson et al. 2021, 2017). Taken together, these variables may help elucidate the associations between minority stress/resilience and sleep.

## 3 Current Study

Understanding variation in sleep quality among gender and sexual minority youth can inform our broader understanding of the health disparities seen in this population compared to their cisgender heterosexual peers. To this end, the current study had two goals. First, we aimed to understand gender and sexual identity group differences in reported trouble falling asleep (one important aspect of sleep quality), considering work suggesting that sleep varies by sex, gender identity, and sexuality (Aernout et al. 2021; Fricke and Sironi 2017; Hershner et al. 2021; Levenson et al. 2021a). The current study builds on this work with a much larger sample (*N* = 11,282) of gender and sexual minority adolescents than in previous studies. We predicted that gender minorities would have more trouble falling asleep than cisgender sexual minorities (Butler et al. 2020). We made no further predictions about differences within groups (e.g., binary vs. nonbinary) because of the lack of sleep research distinguishing among diverse gender identities. We also predicted that plurisexual cisgender youth would have more trouble falling asleep than monosexual cisgender youth (Fricke and Sironi 2017). We focused on cisgender participants for these analyses due to the relative lack of research on sexual identity differences in sleep (or other health variables) among gender minorities.

Our second goal was to understand the association between (a) minority stressors (i.e., violent victimization, bias‐based victimization, and family rejection) and resilience factors (i.e., familial warmth, family acceptance, gender affirming environments, teacher support, trusted adult at school, and presence of a GSA) and (b) trouble falling asleep among gender minority and cisgender sexual minority adolescents, respectively. Some work has suggested minority stress could be one factor in explaining LGBTQ+ sleep disparities (Butler et al. 2020; Kolp et al. 2020). However, little is known about this association among adolescents or the particular factors most related to trouble falling asleep. Consistent with minority stress and social safety theories (L. M. Diamond and Alley 2022; Meyer 2003), we hypothesized that greater levels of minority stress and lower levels of minority resilience would be associated with more trouble falling asleep among gender minority and cisgender sexual minority adolescents. Understanding the role of risk and resilience factors for these two groups separately is important as they likely experience both overlapping and distinct stressors and resilience factors. We expected that variables most closely related to gender/sex, including gender‐based victimization and gender‐affirming school environments, would be particularly related to gender minority adolescents’ sleep quality.

## 4 Materials and Methods

### 4.1 Data Set

The current study's secondary data analysis used data from the *LGBTQ National Teen Survey* (Watson et al. 2021) collected between April and October 2017, which was approved by the Institutional Review Board of the final author's university (H16‐322). Participants were recruited from various social media outlets (e.g., Facebook, Twitter) from both paid and nonpaid posts. Informed consent and a waiver of parental consent were obtained for all participants in the study. This online cross‐sectional survey examined risk and protective factors and related adolescent health outcomes. All eligible participants identified as LGBTQ, spoke English, were 13–17 years of age, and were US residents. Survey respondents who did not fit these criteria were prevented from continuing in the survey to help safeguard data collection. More details on this study's recruitment procedures can be found elsewhere (Watson et al. 2021).

In total, 29,291 people began the survey. Of the 20,306 eligible respondents, an additional 3,006 were excluded because they had answered < 10% of the survey. Among the remaining eligible respondents (*n* = 17,300), a mischievous responder's sensitivity screening was conducted (e.g., to identify misleading or extreme values), open‐ended responses not captured by the screening were analyzed (e.g., to examine suspicious entries), and duplicate surveys were removed (entries that were exact replica responses) leading to the deletion of 175 additional cases (Robinson‐Cimpian 2014). We also removed 33 participants whose gender/sex was not classifiable (see ‘Gender/Sex’ below). Of the remaining respondents, only those who answered a survey question about sleep quality (*N* = 11,282; *M*_*age*_ = 15.6 years, SD = 1.3) were retained. See Table 1 for participant demographics. Data are available upon reasonable request to the authors.

**Table 1:** Sample demographics.

Demographic	*n* (%)
Race/ethnicity	
White	7369 (65)
Biracial or Multiracial	1633 (15)
Hispanic/Latino	1169 (10)
Black	517 (5)
Asian	447 (4)
Native American	53 (1)
Middle Eastern/Arab	39 ( < 1)
Other	36 ( < 1)
Missing	19 ( < 1)
Gender/sex	
Cis girl	4923 (44)
Cis boy	2428 (22)
Nonbinary AFAB	2016 (18)
Trans boy	1449 (13)
Nonbinary AMAB	225 (2)
Trans girl	167 (2)
Questioning AFAB	67 (1)
Questioning AMAB	7 ( < 1)
Sex assigned at birth	
Female	8456 (75)
Male	2826 (25)
Sexual identity	
Gay or Lesbian	4176 (37)
Bisexual	3854 (34)
Pansexual	1567 (14)
Asexual	550 (5)
Queer	501 (4)
Questioning	284 (3)
Straight	180 (2)
Additional sexual identities	170 (2)
Age	
13 years old	844 (8)
14 years old	1678 (15)
15 years old	2320 (21)
16 years old	2963 (26)
17 years old	3477 (31)
Highest education achieved by a caregiver	
Higher than high school degree	8587 (76)
High school degree or lower	2140 (19)
Missing	555 (5)
Sleep location	
Sleeps at home with parents	10,831 (96)
Sleeps somewhere else	438 (4)
Missing	13 ( < 1)

Abbreviations: AFAB, Assigned female at birth; AMAB, Assigned male at birth.

### 4.2 Measures

#### 4.2.1 Gender/Sex and Sexual Identity

Participants were asked two questions to assess gender/sex: their sex assigned at birth (*male* or *female*) and their current gender identity (*male, female, trans male/trans boy, trans female/trans girl, genderqueer/gender non‐conforming*, or *a different identity* which they could write‐in). Participants could choose any number of gender identity options. Using these data, we coded participants into the following categories: cisgender (cis) girls (*n* = 4923), cis boys (*n* = 2428), trans girls (*n* = 167), trans boys (*n* = 1449), nonbinary participants assigned female at birth (nonbinary AFAB; *n* = 2016), nonbinary participants assigned male at birth (nonbinary AMAB; *n* = 225), gender questioning AFAB participants (*n* = 67), and gender questioning AMAB participants (*n* = 7). We coded participants as cisgender if they chose the same sex assigned at birth and gender identity (i.e., *male* and *male* for cisgender boys; *female* and *female* for cisgender girls). We coded participants as trans girls if they chose *male* for sex assigned at birth and *female* and/or *trans female/trans girl* (but not any other options) for gender identity. We coded participants as trans boys if they chose *female* for sex assigned at birth and *male* and/or *trans male/trans boy* (but not any other options) for gender identity. We coded participants as questioning if they who wrote “questioning” or something similar (e.g., ‘confused’) in the write‐in option. We coded participants as nonbinary if they were not questioning and indicated any identities outside of or in addition to male and female through choosing the “genderqueer/gender non‐conforming” option and/or writing in a different identity (e.g., genderfluid, pangender). We decided to separate questioning and nonbinary groups as it was unclear a priori whether adolescents questioning their identities were more similar to binary or nonbinary adolescents, or if they represented their own group altogether. We also further separated gender questioning and nonbinary youth into AMAB or AFAB based on evidence that sex as well as gender differences may contribute to sleep disturbances (Aernout et al. 2021). We dropped the remaining participants (*n* = 33) due to seemingly incommensurate answer choices, such as choosing every option for gender identity.

For **sexual identity**, participants were asked “How do you describe your sexual identity” with the options *Gay or Lesbian, Bisexual, Straight/Heterosexual*, or *Something else*. If they chose *Something else*, they were then presented with a further list of options: *Queer, Pansexual, Asexual, Questioning*, or *Other*. If they chose *Other*, they were asked to type their response. We combined participants who chose *Other* into an “Additional sexualities” category, which included participants who were sexually fluid, demisexual, panromantic, had multiple sexuality labels, and more. See Table 1 for sexual identity across the full sample and Table 2 for sexual identity across gender minority participants, cisgender sexual minority girls, and cisgender sexual minority boys.

**Table 2:** Sexual orientation across gender/sex groups.

Sexual identity	Gender minorities (*n* = 3931)	Sexual minority cis girls (*n* = 4923)	Sexual minority cis boys (*n* = 2428)
	*n* (%)	*n* (%)	*n* (%)
Pansexual	993 (25)	521 (11)	53 (2)
Bisexual	936 (24)	2340 (48)	578 (24)
Gay or Lesbian	871 (22)	1571 (32)	1734 (71)
Queer	334 (9)	151 (3)	16 (1)
Asexual	319 (8)	211 (4)	20 (1)
Straight	180 (5)	0 (0)	0 (0)
Additional sexualities	154 (4)	5 ( < 1)	11 ( < 1)
Questioning	144 (4)	124 (3)	16 (1)

#### 4.2.2 Trouble Falling Asleep

The main outcome variable was trouble falling asleep, which was assessed with a single item: “How often do you have trouble getting to sleep?” on a five‐point scale: *Never* (0), *Sometimes* (1), *About half the time* (2), *Usually* (3), *and Always* (4). Higher scores indicate more trouble falling asleep. Using a single item to assess trouble falling asleep precludes assessments of reliability, though several studies have indicated that single‐item sleep measures are able to predict relevant health outcomes (Atroszko et al. 2015; Cappelleri et al. 2009; Snyder et al. 2018).

#### 4.2.3 Minority Stressors

##### 4.2.3.1 Violent LGBTQ Victimization

Lifetime violent LGBTQ victimization was measured with six items that assessed how often participants experienced verbal, physical, and sexual violence (e.g., *verbal insults*, *punched, kicked, or beaten*) in their lives because of their LGBTQ identities, which has been used in past work with gender and sexual minority youth to show links to mental health outcomes (Baams et al. 2015). Each item used a four‐point scale: *Never* (0), *Once* (1), *Twice* (2), and *3+ Times* (3). For this study, we averaged responses across the six items (α = 0.78). Higher scores indicate more frequent victimization.

##### 4.2.3.2 Bias‐Based Victimization

Participants were asked “How often have you been teased or treated badly by other students at your school because of your:” with eight reasons someone may be victimized. This item has been used in past work with gender and sexual minority youth to show links to health behaviors (Lawrence et al. 2024; Lessard et al. 2020). For the current study, we included the three following forms of bias‐based victimization most relevant to gender and sexual minorities: *gender*, *sexuality*, and *how masculine or feminine I am* (i.e., gender expression). We included each of the three items separately in analyses based on research indicating distinctions between gender, sexuality, and gender expression for victimization (Hoskin 2019). Each item used a five‐point scale: *Never* (0), *Rarely* (1), *Sometimes* (2), *Often* (3), and* Very often* (4). Higher scores indicate more frequent victimization.

##### 4.2.3.3 Family LGBTQ Rejection

Family rejection was measured with four items that assessed how often parents or caregivers rejected participants because of their LGBTQ status (e.g., *taunt or mock you because you are an LGBTQ person*), which has been used in past work with gender and sexual minority youth to show links to health behaviors (Gamarel et al. 2020; Klein and Golub 2016). Each item used a four‐point scale—*Never* (0), *Rarely* (1), *Sometimes* (2), and *Often* (3), and *Very often* (4)—with a *Doesn't apply to me* option was that was treated as missing data. For this study, we averaged responses across the four items (α = 0.89). Higher scores indicate more frequent family rejection.

#### 4.2.4 Minority Resilience Factors

##### 4.2.4.1 Familial Warmth

Familial warmth was measured with three items that assessed how much participants felt their families were warm towards them (e.g., *your family cares about your feelings*), which has been used in past work with gender and sexual minority youth to show links to mental health (Meanley et al. 2021). Each item used a five‐point scale: *Strongly Disagree* (0), *Disagree* (1), *Neither* (2), *Agree* (3), and *Strongly Agree* (4). For this study, we averaged responses across the three items (α = 0.84). Higher scores indicate greater familial warmth.

##### 4.2.4.2 Gender/Sexuality Alliance (GSA) Presence

In line with previous research with gender and sexual minority youth (Lessard et al. 2020; Poteat et al. 2021), participants were asked about the presence (or absence) of a GSA at school. Answer choices were *yes* (coded as 1), *no* (coded as 0), and *do not know* (coded as missing).

##### 4.2.4.3 Gender Affirming School Environments

Participants were also asked questions about their gender affirming environments at school, which have been used in past work with gender minority youth to show links to trans‐related public policies and differences across subgroups of gender minority youth (Renley et al 2022; Simon et al. 2024). For the current study, we included the three items most relevant to gender minority status. First, they were asked about their gender expression: “At school, do you dress and express yourself in a way that matches your gender identity?” on a five‐point scale: *Not at all* (0), *A little* (1), *Somewhat* (2), *Mostly* (3), and *Completely* (4). Second, they were asked about their pronouns: “At school, do adults and students call you by the pronouns (e.g., she, her, hers) that you want to be called?” on a five‐point scale: *Never* (0), *Occasionally* (1), *Sometimes* (2), *Most of the time* (3), and *Always* (4). Third, they were asked about restrooms and locker rooms: “At school, do you use restrooms and locker rooms that match your gender identity?” on a five‐point scale: *Never* (0), *Occasionally* (1), *Sometimes* (2), *Most of the time* (3), and *Always* (4). We included each of the three items separately in the analyses, as gender expression, pronoun use, and restroom use may or may not coincide for an individual based on their social environment. Higher scores indicate more gender affirming school environments.

##### 4.2.4.4 Trusted Adult at School

Consistent with past work with gender and sexual minority youth (Dessel et al. 2017b), having a trusted adult at school was measured using the question: “Is there at least one teacher or other adult in this school that you can talk to if you have a problem?” Answer choices were *yes* (coded as 1), *no* (coded as 0), and *do not know* (coded as missing).

##### 4.2.4.5 Perceived Teacher Support of LGBTQ People

Perceived teacher support was measured with the question “How many of the teachers and staff at your school do you think are supportive of LGBTQ people?” with a four‐point scale—*None of them* (0), *Some of them* (1), *Most of them* (2), and *All of them* (3)—with an *I don't know* option was that was treated as missing data. This question has been used in past work with gender and sexual minority youth in relation to health behaviors (Watson et al. 2021). Higher scores indicate greater perceived support.

##### 4.2.4.6 Family LGBTQ Acceptance

Family acceptance was measured with four items that assessed how often parents or caregivers accepted participants’ LGBTQ status (e.g., *say they like you as you are in regard to being an LGBTQ person*). Each item used a four‐point scale—*Never* (0), *Rarely* (1), *Sometimes* (2), and *Often* (3), and *Very often* (4)—with a *Doesn't apply to me* option was that was treated as missing data. Past work has used these questions with gender and sexual minority youth in conjunction with the four items about family LGBTQ rejection (described above) to show links to mental health (Miller et al. 2020). For this study, we averaged responses across the four items (*α* = 0.81). Higher scores indicate more frequent family acceptance.

#### 4.2.5 Control Variables

##### 4.2.5.1 Depressive Symptoms

Depressive symptoms were measured using a condensed version of the *Kutcher Adolescent Depression Scale* (S. J. Brooks et al. 2003). Participants were asked “Over the last week, how have you been ‘on average’ or ‘usually’ regarding the following items:” with six items (e.g., *Low mood, sadness, feeling blah or down, depressed, just can't be bothered*). We removed two items from the original scale about feeling tired and sleep difficulties to reduce potential overlap with the trouble falling asleep outcome variable. We also removed two items that indexed anxiety symptoms (i.e., feeling nervous and physical feelings of worry)^1^. Each item used a four‐point scale: *Hardly ever* (0), *Much of the time* (1), *Most of the time* (2), and *All of the time* (3). For this study, we averaged responses across the six items (*α* = 0.86). Higher scores indicate greater depressive symptoms. We controlled for depressive symptoms as past work has established a link between this variable and both minority stress/resilience and sleep among LGBTQ+ youth (Baams et al. 2015; Levenson et al. 2021b).

##### 4.2.5.2 Demographic Variables

Participants were asked their birth month and year to assess age (*M*_*age*_ = 15.5 years, SD = 1.3 years). For race/ethnicity, participants were presented with a list of choices and could select all that apply (see Table 1). We dummy coded race/ethnicity for analyses with the largest group, white (non‐Hispanic, non‐Latino), as the reference group. Participants also provided education levels for two parents/primary caregivers using a multiple‐choice question (see Table 1). To have sufficient group sizes for analyses, answers were dichotomized as: *high school education, General Educational Development Test (GED) or less*, and *more than a high school education*. The highest of the caregivers’ scores was included in the analyses as some participants had only one caregiver. Finally, because trouble falling asleep was our main outcome variable, we also controlled for participants’ sleep location, i.e., whether they slept at home with their parents (*n* = 10,831) or somewhere else (*n* = 438). See Table 1 for more demographic details.

### 4.3 Analysis Plan

To assess gender/sex group differences in trouble falling asleep, we conducted a 2 × 3 factorial ANOVA and Tukey post‐hoc tests (H.‐Y. Kim 2015) in IBM SPSS Statistics for Windows (Version 27). We tested the main effects of sex assigned at birth (female and male) and gender identity group (transgender binary, nonbinary, and cisgender) as well as the interaction. We excluded gender questioning adolescents due to insufficient sample size when stratified by sex assigned at birth (*n* = 7 AMAB and *n* = 67 AFAB). Instead, we present descriptive statistics for this group in service of future research. We also conducted two additional one‐way ANOVAs to assess sexual identity group differences for cisgender girls and boys. We included groups with at least 50 participants. Thus, we compared between gay, bisexual, and pansexual boys with a one‐way ANOVA as well as between lesbian, bisexual, queer, pansexual, asexual, and questioning girls with another one‐way ANOVA.

To assess the association between minority stress/resilience and trouble falling asleep, we conducted two regressions: one examining the link among gender minority students controlling for gender identity and sex assigned at birth, and the second examining the links among cisgender sexual minority students, controlling for sex assigned at birth and sexual identity. First we examined variance inflation factors (VIF's) in a regression with all stress and resilience factors predicting trouble falling asleep and determined that there were no issues of multicollinearity (VIF's < 1.98; Kim 2019). We confirmed this by running bivariate correlations between all key variables for gender minorities (Table 3) and cisgender sexual minorities (Table 4), and we found that correlations were low enough to include each variable separately in the regression (*r*'s < 0.56). We then regressed trouble falling asleep onto minority stressors and resilience factors for gender minorities and cisgender sexual minorities separately, controlling for depressive symptoms, age, race/ethnicity, parental education, sleep location, sex assigned at birth (male or female), gender identity for gender minorities only (boy, girl, nonbinary, or questioning, with nonbinary as the reference group), and sexual identity for cisgender sexual minorities only (gay/lesbian, bisexual, queer, pansexual, asexual, questioning, or additional sexual identities, with gay/lesbian as the reference group). We included these control variables as they have each been linked to sleep outcomes (Aernout et al. 2021; Budescu et al. 2024; Fricke and Sironi 2017; Grandner et al. 2016; Hershner et al. 2021). We included the same control variables in each regression (except for gender identity and sexual identity) to maximize their comparability. Within the gender minorities regression, we adjusted analyses for sex assigned at birth and gender identity separately to maximize subsample sizes (i.e., the questioning groups would be too small if also stratified by sex assigned at birth). Both regressions included the same minority stressor and resilience variables except for the gender affirming school environment variables, which were included only in the regression with gender minority participants. We conducted all regressions using Mplus 8.8 (Muthén and Muthén 2010). We used maximum likelihood estimation with standard errors that are robust to non‐normality (MLR) and imputed missing data using maximum likelihood estimates (FIML; Muthén and Muthén 2010).

**Table 3:** Correlations between key study variables for gender minority youth.

Variables	1	2	3	4	5	6	7	8	9	10	11	12	13
1. Trouble falling asleep	—												
2. Violent LGBTQ victimization	0.23**												
3. Familial warmth	−0.24**	−0.27**											
4. Family LGBTQ rejection	−0.19**	−0.27**	0.52**										
5. Family LGBTQ acceptance	−0.11**	0.01	0.45**	0.44**									
6. Gender‐based victimization	0.16**	0.51**	−0.20**	−0.22**	0.05*								
7. Sexuality‐based victimization	0.18**	0.55**	−0.21**	−0.23**	< 0.01	0.61**							
8. Gender expression‐based victimization	0.18**	0.50**	−0.21**	−0.25**	−0.01	0.60**	0.58**						
9. GSA presence	−0.09**	−0.09**	0.09**	0.11**	0.12**	−0.04*	−0.12**	−0.08**					
10. Trusted adult at school	−0.09**	−0.08**	0.17**	0.12**	0.15**	−0.05*	−0.09**	−0.08**	0.33**				
11. Perceived teacher support of LGBTQ people	−0.06**	−0.13**	0.18**	0.22**	0.16**	−0.13**	−0.19**	−0.15**	0.31**	0.33**			
12. School pronoun use	−0.10**	−0.06**	0.18**	0.16**	0.22**	−0.09**	−0.10**	−0.12**	0.19**	0.23**	0.29**		
13. School gender expression	< 0.01	0.06**	0.11**	0.14**	0.21**	0.14**	−0.01	0.01	0.12**	0.14**	0.19**	0.31**	
14. School restroom use	−0.11**	−0.08**	0.17**	0.14**	0.17**	−0.15**	−0.08**	−0.13**	0.09**	0.13**	0.17**	0.54**	0.19**

* *p* < .05

** *p* < .01.

**Table 4:** Correlations between key study variables for cisgender sexual minority youth.

Variables	1	2	3	4	5	6	7	8	9	10
1. Trouble falling asleep	—									
2. Violent LGBTQ victimization	0.21**									
3. Familial warmth	−0.24**	−0.23**								
4. Family LGBTQ rejection	−0.12**	−0.21**	0.43**							
5. Family LGBTQ acceptance	−0.07**	0.02	0.37**	0.38**						
6. Gender‐based victimization	0.16**	0.20**	−0.11**	−0.14**	−0.04**					
7. Sexuality‐based victimization	0.17**	0.58**	−0.20**	−0.20**	0.04**	0.26**				
8. Gender expression‐based victimization	0.14**	0.44**	−0.17**	−0.18**	−0.01	0.24**	0.51**			
9. GSA presence	−0.10**	−0.10**	0.08**	0.08**	0.07**	−0.01	−0.08**	−0.06**		
10. Trusted adult at school	−0.14**	−0.07**	0.19**	0.13**	0.13**	−0.04**	−0.05**	−0.05**	0.27**	
11. Perceived teacher support of LGBTQ people	−0.13**	−0.15**	0.17**	0.19**	0.13**	−0.11**	−0.20**	−0.12**	0.35**	0.34**

* *p* < .05

** *p* < .01.

## 5 Results

### 5.1 Gender/Sex and Sexual Identity Group Differences in Trouble Falling Asleep

**Figure 1:**
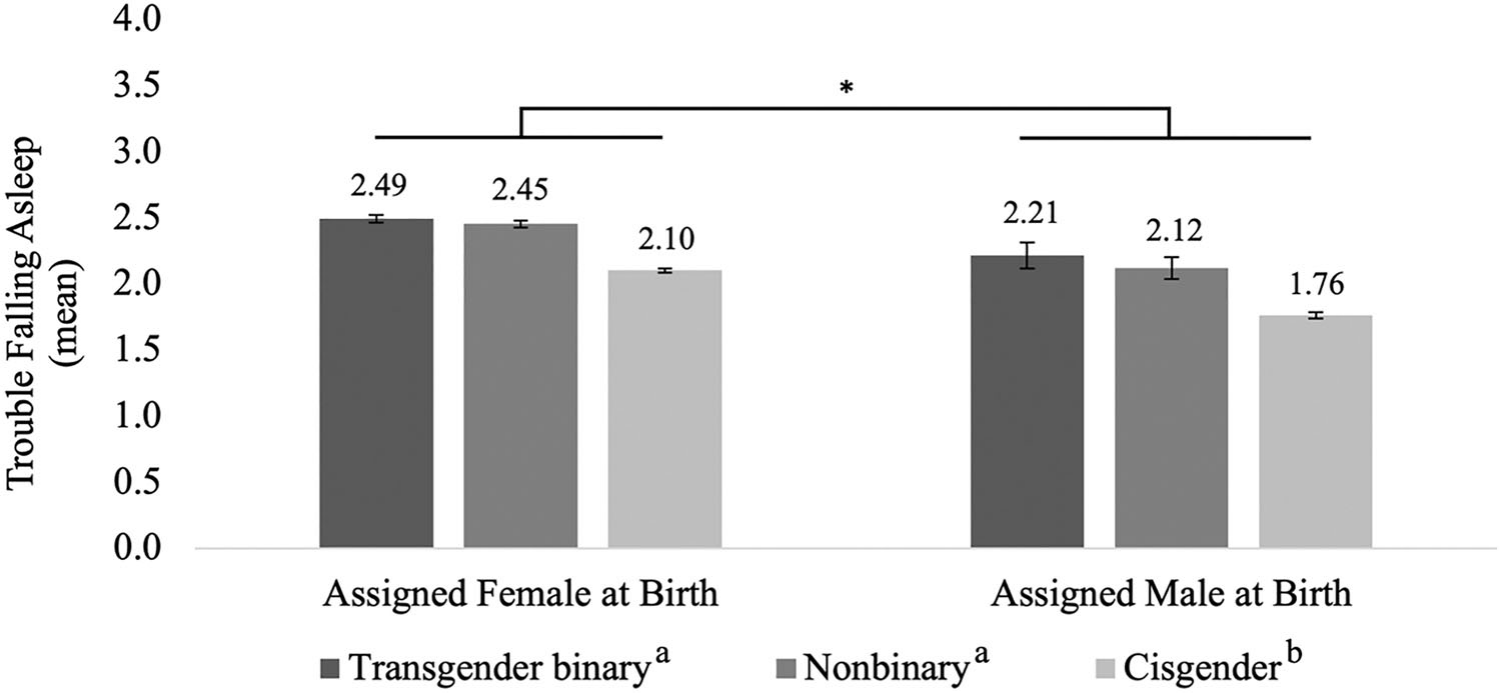
Mean trouble falling asleep across gender/sex groups. *Notes*. “Transgender binary” includes trans boys (assigned female at birth) and trans girls (assigned male at birth). “Nonbinary” includes participants who identified outside of the gender binary. “Cisgender” includes cis girls (assigned female at birth) and cis boys (assigned male at birth). Above each bar is the mean for that gender/sex group. *p < 0.05 significant difference between sex assigned at birth groups. a > b, *p* < 0.05 for gender identity groups. Error bars represent +/– one standard error.

To investigate gender/sex group differences in trouble falling asleep, we conducted a 2 (assigned female at birth [AFAB] and assigned male at birth [AMAB]) x 3 (trans binary, nonbinary, and cisgender) factorial ANOVA (Figure 1). We found a significant main effect of sex assigned at birth with AFAB participants (*M* = 2.25, SD = 1.18) having more trouble falling asleep than AMAB participants (*M* = 1.82, SD = 1.18) with a small effect size, *F*(1, 11,202) = 55.00, *p* < 0.001, η^2^ = 0.005. We also found a significant main effect of gender identity with a small effect size, *F*(2, 11,202) = 62.98, *p* < 0.001, η^2^ = 0.011. Tukey post‐hoc tests revealed that trans binary (*M* = 2.47, SD = 1.18) and nonbinary (*M* = 2.42, SD = 1.17) participants had more trouble falling asleep than cisgender participants (*M* = 1.99, SD = 1.17), *p*'s < 0.001. The interaction between sex assigned at birth and gender identity was not significant, *F*(2, 11,202) = 0.20, *p* = 0.823. Though there were not enough gender questioning youth in our sample to conduct inferential statistics, numerically the mean for AMAB questioning youth (*M* = 2.57, SD = 0.53) was the highest among those assigned male at birth and the mean for AFAB questioning youth (*M* = 2.16, SD = 1.11) were similar to the cisgender girls.

One‐way ANOVA's revealed that sexual identity groups did not significantly differ from one another in trouble falling asleep for either cisgender girls (*F*(5, 4912) = 1.65, *p* = 0.144) or cisgender boys (*F*(2, 2362) = 0.25, *p* = 0.776).

### 5.2 Minority Stress/Resilience on Trouble Falling Asleep

In the next step, we examined the links between minority stress/resilience and trouble falling asleep separately for gender minorities and cisgender sexual minorities, respectively. Starting with risk and resilience factors for sleep among gender minorities, our model accounted for a significant amount of variability in sleep quality, *R* ^*2*^ = 0.17, *p*< 0.001, comparable to previous work examining psychosocial correlates of sleep among adolescents (Chang et al. 2019; Herge et al. 2016; Mang et al. 2023; Martin‐Storey et al. 2024). The model also supported our hypotheses that minority stress factors are linked with more trouble falling asleep and minority resilience factors are linked with less trouble falling asleep (see Table 5). Specifically, controlling for depressive symptoms, age, race/ethnicity, parental education, sex assigned at birth, gender identity, and sleep location, violent LGBTQ victimization (*β* = 0.130, *p* < 0.001) and gender expression‐based victimization (*β* = 0.055, *p* = 0.011) were linked with more trouble falling asleep. Familial warmth (*β* = −0.071, *p* = 0.001) and gender‐matched restroom use (*β* = −0.043, *p* = 0.030) were linked with less trouble falling asleep. No other risk or resilience factors were significantly linked to sleep quality (*p*'s > 0.10).

**Table 5:** Regression results for trouble falling asleep.

	Gender minorities (*n* = 3931)		Cisgender sexual minorities (*n* = 7351)	
Predictor	Standardized *β*	*p* value	Standardized *β*	*p* value
*Stressors*				
Violent LGBTQ victimization	0.130	< 0.001*	0.097	< 0.001*
Gender expression‐based victimization	0.055	0.011*	0.052	< 0.001*
Gender‐based victimization	−0.032	0.151	0.027	0.035*
Sexuality‐based victimization	−0.011	0.630	−0.004	0.782
Family LGBTQ rejection	−0.03	0.129	0.035	0.012*
*Resilience factors*				
Familial warmth	−0.071	0.001*	−0.065	< 0.001*
School restroom use	−0.043	0.030*		
GSA presence	−0.029	0.113	−0.030	0.018*
Trusted adult at school	−0.007	0.717	−0.026	0.049*
School pronoun use	−0.004	0.850		
School gender expression	0.013	0.499		
Perceived teacher support of LGBTQ people	0.031	0.100	−0.013	0.346
Family LGBTQ acceptance	−0.001	0.950	0.003	0.806
*Control variables*				
Depressive symptoms	0.257	< 0.001*	0.345	< 0.001*
Age	−0.071	< 0.001*	−0.065	< 0.001*
Assigned female at birth^a^	0.063	0.003*	0.089	< 0.001*
Caregivers’ highest education^b^	−0.012	0.451	0.010	0.391
Sleep location^c^	−0.001	0.999	0.013	0.191
*Gender identity* d				
Boy	−0.033	0.048*		
Questioning	−0.014	0.318		
Girl	0.008	0.688		
*Race/ethnicity* e				
Black/African American	−0.053	0.001*	−0.040	< 0.001*
Asian/Pacific Islander	−0.030	0.060	−0.032	0.004*
Latino/Hispanic/Mexican American	0.014	0.341	< 0.001	0.974
Native American/Alaska Native	−0.008	0.430	0.012	0.285
Middle Eastern/Arab	0.010	0.476	−0.006	0.544
Additional race/ethnicities	−0.007	0.670	−0.005	0.596
Biracial/multiracial	0.005	0.731	0.013	0.226
*Sexual identity* f				
Asexual			0.022	0.041*
Additional sexual identities			0.014	0.176
Bisexual			0.009	0.457
Questioning			−0.004	0.723
Queer			−0.003	0.788
Pansexual			< 0.001	0.970

Model fit *R* ^*2*^ = 0.169, *p* < 0.001 *R* ^*2*^ = 0.222, *p* < 0.001.

*Notes*. *p < 0.05.

^a^ reference group is assigned male at birth.

^b^ 0 = *high school education or less*, 1 = *more than high school education.*

^c^ 0 = *sleeps at home with parents/primary caregivers*, 1 = *sleeps anywhere else.*

^d^ reference group is nonbinary.

^e^ reference group is white (non‐Hispanic, non‐Latino).

^f^ reference group is gay/lesbian.

For cis sexual minorities, our model accounted for a significant amount of variability in sleep quality, *R* ^*2*^ = 0.22, *p* < 0.001, again comparable to previous work (Chang et al. 2019; Herge et al. 2016; Mang et al. 2023; Martin‐Storey et al. 2024). The model also supported our hypotheses that minority stress factors are linked with more trouble falling asleep and minority resilience factors are linked with less trouble falling asleep (see Table 5). Specifically, controlling for depressive symptoms, age, race/ethnicity, parental education, sex assigned at birth, sexual identity, and sleep location, violent LGBTQ victimization (*β* = 0.097, *p* < 0.001), family rejection (*β* = 0.035, *p* = 0.012), gender‐based victimization (*β* = 0.027, *p* = 0.035), and gender expression‐based victimization (*β* = 0.052, *p* < 0.001) were linked with more trouble falling asleep. Familial warmth (*β* = −0.065, *p* < 0.001), presence of a GSA (*β* = −0.030, *p* = 0.018), and trusted adult presence (*β* = −0.026, *p* = 0.049) were linked with less trouble falling asleep. No other risk or resilience factors were significantly linked to sleep quality (*p*'s > 0.35).

## 6 Discussion

Sleep quality is central to adolescent well‐being, with research increasingly suggesting that sleep health may be important for understanding disparities between LGBTQ+ youth and their heterosexual cisgender peers (Dai et al. 2020; Martin‐Storey et al. 2020; Nagata et al. 2023). Our findings underscore how minority stressors reflective of threats to social safety such as violent victimization and gender expression‐based victimization, and minority resilience factors associated with social safety such as familial warmth and gender‐segregated restroom use were related to less trouble falling asleep for both gender and sexual minority youth. Gender‐based victimization, family rejection, presence of a GSA, and having a trusted adult at school were also related to trouble falling asleep for cisgender sexual minority youth. Our hypothesis that gender minority youth would have more trouble falling asleep than cisgender youth was also supported via small but significant differences across gender/sex, and we additionally found that youth assigned female at birth reported more trouble falling asleep than youth assigned male at birth. Our hypothesis that plurisexual cisgender youth would have worse sleep than monosexual cisgender youth, however, was not supported. These findings are discussed in greater detail below.

The gender/sex group differences we found suggest that both sex assigned at birth and gender identity matter for sleep. AFAB participants in our sample had more trouble falling asleep than AMAB participants, which aligns with research with cisgender populations (Aernout et al. 2021). These differences may be related to physiological processes generally linked to sex assigned at birth, such as hormonal cycles (Krishnan and Collop 2006), and/or psychosocial processes linked to gender socialization, such as rumination or other stressors experienced by these youth (Clancy et al. 2020; Jose and Brown 2008). However, gender identity matters as well. Both trans binary (girls and boys) and nonbinary participants had more trouble falling asleep than the cis sexual minority participants. Thus, among LGBTQ+ youth, those minoritized on the basis of gender seem most at risk for sleep problems. This finding aligns with past work showing that gender minority youth often have worse health and psychosocial outcomes than cisgender sexual minorities (Delozier et al. 2020). However, while differences across gender/sex groups were significant, the small effect sizes (η^2^'s < 0.012) suggest that group characteristics related to sex assigned at birth and gender identity may be limited in their association with trouble falling asleep among LGBTQ+ adolescents.

The current study also highlighted the role of minority stressors for sleep health among gender and sexual minority youth, specifically underscoring the relevance of social safety. An extensive literature links victimization and family rejection to health outcomes for minoritized youth (Bouris et al. 2010; Delozier et al. 2020), so it is unsurprising that we found links between these stressors and trouble falling asleep in this sample. Violent victimization, bias‐based victimization, and family rejection represent the loss of social protection. Thus, disruptions in sleep quality may be explained by minority stress, and particularly minority stressors that lead to disruptions in adolescents’ social safety (L. M. Diamond and Alley 2022; Meyer 2003).

Across both groups, gender expression‐based victimization in particular was related to more trouble falling asleep. Compared to gender identity and sexuality (each of which may be concealed by youth), gender expression is the most visible component of gender included in the current study, and unlike gender identity or sexuality, may be more difficult to selectively conceal. Combined with research showing that sexuality is often regulated through the policing of gender expression and vice versa (Hoskin 2019; Pascoe 2005), these results suggest that youth with nonconforming gender expressions may be particularly vulnerable to victimization and subsequent sleep disruptions.

Resilience factors are often overlooked in research on minority stress, yet we found important links between resilience and sleep. Familial warmth's association with less trouble falling asleep emphasizes the central role of family functioning for minoritized adolescents’ health, as has been highlighted in past work (e.g., Meanley et al. 2021). The association among sexual minorities between better sleep and the presence of both a GSA and a trusted adult at school further highlight supportive school environments as important for health (Dessel et al. 2017a; Toomey et al. 2011). These findings may reflect how, from a social safety perspective (L. M. Diamond and Alley 2022), connection to one's family and safe spaces at school can provide feelings of belonging and protection that improve sleep health. These results suggest that approaches designed to improve minoritized adolescents’ family and school lives may have implications for sleep and should be considered in future research. It is unclear, however, why we did not find similar associations between school environments and sleep among gender minority youth. It may be that with fewer gender minorities in the sample, these associations were underpowered. It also may be that experiences specific to gender minoritization prevent beneficial effects of ostensibly affirming school environments. For example, GSA's can sometimes center sexuality‐related issues at the detriment of gender‐related issues, and gender minority people can experience cissexism even from cisgender sexual minorities, thereby limiting a GSA's impact on feelings of social safety (Galupo et al. 2014; Sutherland 2019). Having a trusted adult at school may also not be as impactful for gender minority youth amidst a broader environment of cissexism, considering the majority of Americans hold beliefs that disaffirm gender diversity (Parker et al. 2022).

We also found that gender‐matched school restroom and changing room use is related to less trouble falling asleep for gender minority participants. Issues of restrictive restroom access negatively affect the mental health of gender minority youth (Price‐Feeney et al. 2021), and our results suggest that restrictive policies and norms may work their way “under the skin” through their association with sleep quality. Further research is needed to establish if gender‐matched restroom use itself is linked with sleep quality, or if this variable tracks the effects of related public policies that explicitly regulate who can access which gender‐segregated restrooms and changing rooms (Kurth et al. 2021). In any case, providing gender minority adolescents with gender‐affirming environments could help address sleep disparities.

### 6.1 Limitations and Future Directions

The current study has numerous strengths including the relatively large sample size, the inclusion of transgender, cisgender, binary, nonbinary, and gender questioning identities, and the measurement of multiple stress and resilience factors specific to gender and sexual minority experiences. Despite these strengths, the findings should be interpreted in light of several limitations. First, trouble falling asleep was measured with a single subjective item without a clear timeframe, as was also the case for the bias‐based victimization questions. Though single sleep items have been shown to be effective predictors of health (Atroszko et al. 2015; Cappelleri et al. 2009; Snyder et al. 2018), in the future, using scales that encompass multiple aspects of sleep quality and victimization with a clear timeframe (Buysse et al. 1989) and/or objective measures of sleep may provide more rigorous measurements of sleep quality and other variables (Tavernier and Willoughby 2014).

Second, the beta values for the regressions indicate small effect sizes (Cohen 1988) and our models explained only 17%–22% of the variance in trouble falling asleep. As sleep is an incredibly complex biopsychosocial process, it is not necessarily surprising that our variables did not predict a large amount of variance in sleep quality. Indeed, other studies of the social determinants of sleep find comparative effect sizes (e.g., Aggarwal et al. 2019; Mang et al. 2023). Nevertheless, these small effect sizes warrant caution in interpreting the practical application of our results and future work with more comprehensive measures of sleep may reveal stronger associations between minority stress/resilience and sleep. One potential source of variance missing in our study is medical transition status, since the *2017 LGBTQ National Teen Survey* (Watson et al. 2021) did not ask about puberty blockers or hormone therapy. The scant research on this association in adults generally finds little to no association between hormone therapy and sleep, and one study found that trans youth who had engaged in medical transition had better sleep outcomes than those who had not (Gavidia et al. 2022; Morssinkhof et al. 2023). Thus, future work could unpack the complex relationships between medical transition and sleep for gender minority youth.

Further, the cross‐sectional nature of our data did not allow for investigating the direction of the relation between trouble falling asleep and stress/resilience, which could be addressed with longitudinal studies. Longitudinal work could also help elucidate the consequences of poor sleep quality and minority stress/resilience. Poor sleep quality has been related to suicidal ideation via affective reactivity to negative interpersonal events for youth at high risk for suicide and depression (Hamilton et al. 2022). Future work should therefore not only examine the links between minority stress/resilience factors and sleep prospectively, but should also examine how these processes play into the well‐documented mental health disparities among gender and sexual minority youth (Delozier et al. 2020; Hafeez et al. 2017; Lucassen et al. 2017).

Finally, characteristics of the sample also limit the generalizability of our findings. Though we presented descriptive statistics for questioning youth, this subsample was not large enough to draw sufficient conclusions about their relative sleep quality, indicating that future studies should intentionally recruit this understudied group. Additionally, the sample was moderately diverse by race/ethnicity, though some groups were underrepresented (e.g., Black youth, Asian/Pacific Islander youth). The sample was also disproportionately high in socioeconomic status (assessed via parental education) compared to the general U.S. population (United States Census Bureau 2022). Perhaps this is due to the online recruitment method, which, at least with transgender adults, is more likely to garner a sample that is more predominantly white and higher in socioeconomic status than in‐person methods (Reisner et al. 2014). These findings should be replicated in more diverse and in‐person samples. These findings should also be replicated with a newer sample. Much has changed socio‐politically in the lives of LGBTQ youth since 2017 when the current study's data were collected. Across many US states and the federal government, there has been a massive increase in policies proposed and passed that directly target LGBTQ youth, especially transgender youth (American Civil Liberties Union 2025). This may have changed the landscape of the minority stressors and resilience factors relevant in youth's lives, as well as their relative importance for health. Our results suggest that future work with gender and sexual minority youth should include disruptions in sleep as one potential impact of this political environment.

### 6.2 Implications for Clinical Practice

Our results have several implications for clinical practice. In general, clinicians working with minoritized youth might find it beneficial to discuss sleep with their clients. Results suggest therapeutic techniques for sleep, like sleep hygiene education and cognitive behavioral therapy (Morin et al. 2007), would be particularly beneficial to gender minoritized youth and those assigned female at birth as they had the most trouble falling asleep, though with small effect sizes for group differences, these techniques would likely benefit all groups. Further, if clients are experiencing threats to social safety, clinicians may expect negative sleep consequences. Our findings also point to the relevance of addressing the family context for understanding sleep outcomes. Thus, family therapy may be especially beneficial for minoritized youth's sleep quality, considering the protective nature of familial warmth and the risk associated with family rejection for cisgender sexual minority youth. Family therapy techniques such as attachment‐based and narrative therapy and have been shown, at least in case studies, to be related to reduced suicidality among gender and sexual minority youth (G. M. Diamond et al. 2012; Jordan 2020; Russon et al. 2022). Finally, as having a trusted adult at school was linked with less trouble falling asleep, at least for cisgender sexual minority youth, clinicians may encourage young clients to seek out these adults in their schools. Clinicians who work in schools (e.g., school nurses, social workers, psychologists) can also themselves be that trusted adult at school who can provide feelings of safety and belonging for LGBTQ+ youth.

## 7 Conclusions

Our study provides evidence that minority stressors such as exposure to LGBTQ victimization, gender‐expression based victimization, and family rejection, and resilience factors such as familial warmth, gender‐matched restroom use, GSA presence, and having a trusted adult in the school are important experiences in understanding sleep health among gender and sexual minority youth, providing avenues for future research exploring the underappreciated role of sleep in minoritized health disparities. Our results also support interventions at interpersonal (e.g., family functioning) and institutional (e.g., GSA presence) levels in service of ameliorating the biopsychosocial harms that a cisheteronormative society inflicts upon LGBTQ+ youth. In particular, our findings highlight the relevance of focusing on social safety‐related risk and resilience factors to better understand sleep health in particular. Overall, the current study is an important step in understanding the complex associations between stress, resilience, and health for gender and sexual minority adolescents.

## Conflicts of Interest

The authors declare no conflicts of interest.

## Data Availability

The data that support the findings of this study are available from the corresponding author upon reasonable request.

## Endnotes

1 The researchers in the larger study also removed one item about suicidality due to ethical concerns as the participants provided a waiver of parental consent.

## References

[jad12520-bib-0001] Aernout, E. , I. Benradia , J.‐B. Hazo , et al. 2021. “International Study of the Prevalence and Factors Associated With Insomnia in the General Population.” Sleep Medicine 82: 186–192. 10.1016/j.sleep.2021.03.028. 33957414

[jad12520-bib-1002] Aggarwal, B. , N. Makarem , M. Liao , Z. Mayat , S. Byun , and E.‐G. Giardina . 2019. “Psychosocial Factors are Strongly Associated With Sleep Disturbances and Evening Chronotype Among Diverse Women: Evidence From the AHA Go Red for Women Strategically Focused Research Network.” Supplement, Circulation 139, no. Suppl_1. 10.1161/circ.139.suppl_1.P281.

[jad12520-bib-0002] American Civil Liberties Union . (2025). Mapping Attacks on LGBTQ Rights in U.S. State Legislatures in 2025. https://www.aclu.org/legislative-attacks-on-lgbtq-rights-2025.

[jad12520-bib-0005] Arslan, G. 2021. “School Belongingness, Well‐Being, and Mental Health Among Adolescents: Exploring the Role of Loneliness.” Australian Journal of Psychology 73, no. 1: 70–80. 10.1080/00049530.2021.1904499.

[jad12520-bib-0006] Atroszko, P. , P. Bagińska , M. Mokosińska , and B. Atroszko . 2015. “Validity and Reliability of Single‐Item Self‐Report Measures of General Quality of Life, General Health and Sleep Quality.” Comparative European Research 2: 207–211.

[jad12520-bib-0007] Auer, M. K. , A. Liedl , J. Fuss , et al. 2017. “High Impact of Sleeping Problems on Quality of Life in Transgender Individuals: A Cross‐Sectional Multicenter Study.” PLoS One 12, no. 2: e0171640. 10.1371/journal.pone.0171640. 28199359 PMC5310898

[jad12520-bib-0008] Baams, L. , A. H. Grossman , and S. T. Russell . 2015. “Minority Stress and Mechanisms of Risk for Depression and Suicidal Ideation Among Lesbian, Gay, and Bisexual Youth.” Developmental Psychology 51, no. 5: 688–696. 10.1037/a0038994. 25751098 PMC4412799

[jad12520-bib-0009] Biddle, D. J. , D. F. Hermens , T. Lallukka , M. Aji , and N. Glozier . 2019. “Insomnia Symptoms and Short Sleep Duration Predict Trajectory of Mental Health Symptoms.” Sleep Medicine 54: 53–61. 30529778 10.1016/j.sleep.2018.10.008

[jad12520-bib-0010] Bouris, A. , V. Guilamo‐Ramos , A. Pickard , et al. 2010. “A Systematic Review of Parental Influences on the Health and Well‐Being of Lesbian, Gay, and Bisexual Youth: Time for a New Public Health Research and Practice Agenda.” The Journal of Primary Prevention 31, no. 5–6: 273–309. 10.1007/s10935-010-0229-1. 21161599

[jad12520-bib-0011] Bowen, A. E. , S. Staggs , J. Kaar , N. Nokoff , and S. L. Simon . 2021. “Short Sleep, Insomnia Symptoms, and Evening Chronotype Are Correlated With Poorer Mood and Quality of Life in Adolescent Transgender Males.” Sleep Health 7, no. 4: 445–450. 10.1016/j.sleh.2021.03.008. 33875385 PMC8384662

[jad12520-bib-0012] Brooks, S. J. , S. P. Krulewicz , and S. Kutcher . 2003. “The Kutcher Adolescent Depression Scale: Assessment of Its Evaluative Properties Over the Course of an 8‐week Pediatric Pharmacotherapy Trial.” Journal of Child and Adolescent Psychopharmacology 13, no. 3: 337–349. 10.1089/104454603322572679. 14642022

[jad12520-bib-0013] Brooks, V. R. 1981. Minority Stress and Lesbian Women. Lexington Books.

[jad12520-bib-0014] Budescu, M. , A. Reid , A. Sisselman‐Borgia , N. Holbrook , D. Valera , and G. C. Torino . 2024. “Sleep and Mental Health Among Youth Experiencing Homelessness: A Retrospective Pilot Diary Study.” Sleep Health 10, no. 1: 54–59. 10.1016/j.sleh.2023.10.003. 37989625

[jad12520-bib-0015] Butler, E. S. , E. McGlinchey , and R. P. Juster . 2020. “Sexual and Gender Minority Sleep: A Narrative Review and Suggestions for Future Research.” Journal of Sleep Research 29, no. 1. 10.1111/jsr.12928. 31626363

[jad12520-bib-0016] Buysse, D. J. , C. F. Reynolds , T. H. Monk , S. R. Berman , and D. J. Kupfer . 1989. “The Pittsburgh Sleep Quality Index: A New Instrument for Psychiatric Practice and Research.” Psychiatry Research 28, no. 2: 193–213. 10.1016/0165-1781(89)90047-4. 2748771

[jad12520-bib-0017] Cappelleri, J. C. , A. G. Bushmakin , A. M. McDermott , A. B. Sadosky , C. D. Petrie , and S. Martin . 2009. “Psychometric Properties of a Single‐Item Scale to Assess Sleep Quality Among Individuals With Fibromyalgia.” Health and Quality of Life Outcomes 7, no. 1: 54. 10.1186/1477-7525-7-54. 19534799 PMC2706811

[jad12520-bib-0018] Cappuccio, F. P. , D. Cooper , L. D'Elia , P. Strazzullo , and M. A. Miller . 2011. “Sleep Duration Predicts Cardiovascular Outcomes: A Systematic Review and Meta‐Analysis of Prospective Studies.” European Heart Journal 32, no. 12: 1484–1492. 21300732 10.1093/eurheartj/ehr007

[jad12520-bib-0019] Carskadon, M. A. 2011. “Sleep in Adolescents: The Perfect Storm.” Pediatric Clinics of North America 58, no. 3: 637–647. 10.1016/j.pcl.2011.03.003. 21600346 PMC3130594

[jad12520-bib-0020] Chang, L.‐Y. , C.‐C. Wu , L. N. Lin , H.‐Y. Chang , and L.‐L. Yen . 2019. “Age and Sex Differences in the Effects of Peer Victimization on Depressive Symptoms: Exploring Sleep Problems as a Mediator.” Journal of Affective Disorders 245: 553–560. 30439680 10.1016/j.jad.2018.11.027

[jad12520-bib-0021] Clancy, F. , A. Prestwich , L. Caperon , A. Tsipa , and D. B. O'Connor . 2020. “The Association Between Worry and Rumination With Sleep in Non‐Clinical Populations: A Systematic Review and Meta‐Analysis.” Health Psychology Review 14, no. 4: 427–448. 10.1080/17437199.2019.1700819. 31910749

[jad12520-bib-0022] Clark, K. A. , K. Schafer , N. M. Tran , L. Trautman , and T. McKay . 2023. “The Role of Sleep Duration in Suicide Risk Among Sexual and Gender Minority Adolescents.” Preventive Medicine 175: 107698. 10.1016/j.ypmed.2023.107698. 37704179 PMC10591716

[jad12520-bib-0023] Cohen, J. 1988. Statistical Power Analysis for the Behavioral Sciences. Erlbaum Associates.

[jad12520-bib-0024] Dai, H. , and J. Hao . 2019. “Sleep Deprivation and Chronic Health Conditions Among Sexual Minority Adults.” Behavioral Sleep Medicine 17, no. 3: 254–268. 10.1080/15402002.2017.1342166. 28657361

[jad12520-bib-0025] Dai, H. , D. G. Ingram , and J. B. Taylor . 2020. “Hierarchical and Mediation Analysis of Disparities in Very Short Sleep Among Sexual Minority Youth in the U.S., 2015.” Behavioral Sleep Medicine 18, no. 4: 433–446. 10.1080/15402002.2019.1607738. 31068017

[jad12520-bib-0026] Delozier, A. M. , R. C. Kamody , S. Rodgers , and D. Chen . 2020. “Health Disparities in Transgender and Gender Expansive Adolescents: A Topical Review From a Minority Stress Framework.” Journal of Pediatric Psychology 45, no. 8: 842–847. 10.1093/jpepsy/jsaa040. 32626901

[jad12520-bib-0027] Dessel, A. B. , A. Kulick , L. J. Wernick , and D. Sullivan . 2017a. “The Importance of Teacher Support: Differential Impacts by Gender and Sexuality.” Journal of Adolescence 56, no. 1: 136–144. 10.1016/j.adolescence.2017.02.002. 28231485

[jad12520-bib-0028] Dessel, A. B. , A. Kulick , L. J. Wernick , and D. Sullivan . 2017b. “The Importance of Teacher Support: Differential Impacts by Gender and Sexuality.” Journal of Adolescence 56, no. 1: 136–144. 10.1016/j.adolescence.2017.02.002. 28231485

[jad12520-bib-0029] Diamond, G. M. , G. S. Diamond , S. Levy , C. Closs , T. Ladipo , and L. Siqueland . 2012. “Attachment‐Based Family Therapy for Suicidal Lesbian, Gay, and Bisexual Adolescents: A Treatment Development Study and Open Trial With Preliminary Findings.” Psychotherapy 49, no. 1: 62–71. 10.1037/a0026247. 22181026

[jad12520-bib-0030] Diamond, L. M. , and J. Alley . 2022. “Rethinking Minority Stress: A Social Safety Perspective on the Health Effects of Stigma in Sexually‐Diverse and Gender‐Diverse Populations.” Neuroscience and Biobehavioral Reviews 138, no. July 2022: 104720. 10.1016/j.neubiorev.2022.104720. 35662651

[jad12520-bib-0031] Eom, Y.‐J. , H. Lee , R. Kim , S. Choo , H. Yi , and S.‐S. Kim . 2022. “Discrimination Keeps Transgender People Awake at Night: A Nationwide Cross‐Sectional Survey of 583 Transgender Adults in South Korea.” Sleep Health 8, no. 6: 580–586. 10.1016/j.sleh.2022.06.012. 36050274

[jad12520-bib-0032] Fricke, J. , and M. Sironi . 2017. “Dimensions of Sexual Orientation and Sleep Disturbance Among Young Adults.” Preventive Medicine Reports 8: 18–24. 10.1016/j.pmedr.2017.07.008. 28831369 PMC5555087

[jad12520-bib-0033] Galupo, M. P. , S. B. Henise , and K. S. Davis . 2014. “Transgender Microaggressions in the Context of Friendship: Patterns of Experience Across Friends’ Sexual Orientation and Gender Identity.” Psychology of Sexual Orientation and Gender Diversity 1, no. 4: 461–470. 10.1037/sgd0000075.

[jad12520-bib-0034] Gamarel, K. E. , R. J. Watson , R. Mouzoon , C. W. Wheldon , J. N. Fish , and N. L. Fleischer . 2020. “Family Rejection and Cigarette Smoking Among Sexual and Gender Minority Adolescents in the USA.” International journal of behavioral medicine 27, no. 2: 179–187. 10.1007/s12529-019-09846-8. 31925674 PMC7124998

[jad12520-bib-0035] Gavidia, R. , D. G. Whitney , S. Hershner , E. M. Selkie , R. Tauman , and G. L. Dunietz . 2022. “Gender Identity and Transition: Relationships With Sleep Disorders in US Youth.” Journal of Clinical Sleep Medicine 18, no. 11: 2553–2559. 10.5664/jcsm.10158. 35912700 PMC9622987

[jad12520-bib-0036] Goldstein, T. R. , and P. L. Franzen . 2022. “A Comprehensive Review of the Literature on Sleep Difficulties and Suicidality in Youth to Inform an Integrative Developmental Model and Future Directions.” Current Sleep Medicine Reports 8, no. 1: 1–19. 10.1007/s40675-022-00222-9. 36274826 PMC9586157

[jad12520-bib-0037] Grandner, M. A. , N. J. Williams , K. L. Knutson , D. Roberts , and G. Jean‐Louis . 2016. “Sleep Disparity, Race/Ethnicity, and Socioeconomic Position.” Sleep Medicine 18: 7–18. 10.1016/j.sleep.2015.01.020. 26431755 PMC4631795

[jad12520-bib-0038] Hafeez, H. , M. Zeshan , M. A. Tahir , N. Jahan , and S. Naveed . 2017. “Health Care Disparities Among Lesbian, Gay, Bisexual, and Transgender Youth: A Literature Review.” Cureus 9, no. 4: e1184. 10.7759/cureus.1184. 28638747 PMC5478215

[jad12520-bib-0039] Hamilton, J. L. , A. Tsypes , J. Zelazny , et al. 2023. “Sleep Influences Daily Suicidal Ideation Through Affective Reactivity to Interpersonal Events Among High‐Risk Adolescents and Young Adults.” Journal of Child Psychology and Psychiatry, and Allied Disciplines 64: 27–38. 10.1111/jcpp.13651. 35778912 PMC9876533

[jad12520-bib-0040] Herge, W. M. , A. M. La Greca , and S. F. Chan . 2016. “Adolescent Peer Victimization and Physical Health Problems.” Journal of Pediatric Psychology 41, no. 1: 15–27. 26050245 10.1093/jpepsy/jsv050 PMC4902864

[jad12520-bib-0041] Hershner, S. , E. Jansen , R. Gavidia , L. Matlen , M. Hoban , and G. L. Dunietz . 2021. “Associations Between Transgender Identity, Sleep, Mental Health and Suicidality Among a North American Cohort of College Students.” Nature and Science of Sleep 13: 383–398. 10.2147/nss.s286131. PMC7982442 33762860

[jad12520-bib-0042] Hoskin, R. A. 2019. “Femmephobia: The Role of Anti‐Femininity and Gender Policing in LGBTQ+ People's Experiences of Discrimination.” Sex Roles 81: 686–703. 10.1007/s11199-019-01021-3.

[jad12520-bib-0043] Huang, Y. , P. Li , Z. Lai , et al. 2018. “Role of Sleep Quality in Mediating the Relationship Between Sexual Minority Status and Suicidal Behavior Among Chinese Adolescents.” Psychology Research and Behavior Management 11: 607–615. 10.2147/PRBM.S186586. 30573996 PMC6292244

[jad12520-bib-0044] Jordan, F. 2020. “Changing the Narrative for LGBTQ Adolescents: A Literature Review and Call for Research Into Narrative Therapy to Improve Family Acceptance of LGBTQ Teens.” Counseling and Family Therapy Scholarship Review 3, no. 1: 6. 10.53309/RAQX6953.

[jad12520-bib-0045] Jose, P. E. , and I. Brown . 2008. “When Does the Gender Difference in Rumination Begin? Gender and Age Differences in the Use of Rumination by Adolescents.” Journal of Youth and Adolescence 37, no. 2: 180–192. 10.1007/s10964-006-9166-y.

[jad12520-bib-0046] Kelleher, C. 2009. “Minority Stress and Health: Implications for Lesbian, Gay, Bisexual, Transgender, and Questioning (LGBTQ) Young People.” Counselling Psychology Quarterly 22, no. 4: 373–379. 10.1080/09515070903334995.

[jad12520-bib-0047] Kim, H.‐Y. 2015. “Statistical Notes for Clinical Researchers: *Post‐Hoc* Multiple Comparisons.” Restorative Dentistry & Endodontics 40, no. 2: 172–176. 10.5395/rde.2015.40.2.172. 25984481 PMC4432262

[jad12520-bib-0048] Kim, J. H. 2019. “Multicollinearity and Misleading Statistical Results.” Korean Journal of Anesthesiology 72, no. 6: 558–569. 10.4097/kja.19087. 31304696 PMC6900425

[jad12520-bib-0049] Klein, A. , and S. A. Golub . 2016. “Family Rejection as a Predictor of Suicide Attempts and Substance Misuse Among Transgender and Gender Nonconforming Adults.” LGBT Health 3, no. 3: 193–199. 10.1089/lgbt.2015.0111. 27046450

[jad12520-bib-0050] Kolp, H. , S. Wilder , C. Andersen , et al. 2020. “Gender Minority Stress, Sleep Disturbance, and Sexual Victimization in Transgender and Gender Nonconforming Adults.” Journal of Clinical Psychology 76, no. 4: 688–698. 10.1002/jclp.22880. 31626334

[jad12520-bib-0051] Krishnan, V. , and N. A. Collop . 2006. “Gender Differences in Sleep Disorders.” Current Opinion in Pulmonary Medicine 12, no. 6: 383–389. 10.1097/01.mcp.0000245705.69440.6a. 17053485

[jad12520-bib-0052] Kurth, A. E. , J. A. Puckett , and K. D. Anderson‐Carpenter . 2021. “Legislation Restricting Access to Public Restrooms and Changing Facilities for Transgender Individuals in Texas (US): A Qualitative Analysis of Testimony.” International Journal of Transgender Health 22: 440–453. 10.1080/26895269.2021.1905580. 37808527 PMC10553368

[jad12520-bib-0053] Lawrence, S. E. , R. J. Watson , H. M. Eadeh , C. Brown , R. M. Puhl , and M. E. Eisenberg . 2024. “Bias‐Based Bullying, Self‐Esteem, Queer Identity Pride, and Disordered Eating Behaviors Among Sexually and Gender Diverse Adolescents.” International Journal of Eating Disorders 57, no. 2: 303–315. 10.1002/eat.24092. 37990394 PMC10922269

[jad12520-bib-0054] Lessard, L. M. , R. J. Watson , and R. M. Puhl . 2020. “Bias‐Based Bullying and School Adjustment Among Sexual and Gender Minority Adolescents: The Role of Gay‐Straight Alliances.” Journal of Youth and Adolescence 49, no. 5: 1094–1109. 10.1007/s10964-020-01205-1. 32246306

[jad12520-bib-0055] Levenson, J. C. , B. C. Thoma , J. L. Hamilton , S. Choukas‐Bradley , and R. H. Salk . 2021a. “Sleep Among Gender Minority Adolescents.” Sleep 44, no. 3: zsaa185. 10.1093/sleep/zsaa185. 32949142 PMC7953209

[jad12520-bib-0056] Levenson, J. C. , B. C. Thoma , J. L. Hamilton , S. Choukas‐Bradley , and R. H. Salk . 2021b. “Sleep Among Gender Minority Adolescents.” Sleep 44, no. 3: zsaa185. 10.1093/sleep/zsaa185. 32949142 PMC7953209

[jad12520-bib-0057] Li, P. , Y. Huang , L. Guo , et al. 2017. “Is Sexual Minority Status Associated With Poor Sleep Quality Among Adolescents? Analysis of a National Cross‐Sectional Survey in Chinese Adolescents.” BMJ Open 7, no. 12: e017067. 10.1136/bmjopen-2017-017067. PMC5770949 29282258

[jad12520-bib-0058] Lucassen, M. F. , K. Stasiak , R. Samra , C. M. Frampton , and S. N. Merry . 2017. “Sexual Minority Youth and Depressive Symptoms or Depressive Disorder: A Systematic Review and Meta‐Analysis of Population‐Based Studies.” The Australian and New Zealand Journal of Psychiatry 51, no. 8: 774–787. 10.1177/0004867417713664. 28565925

[jad12520-bib-0059] Mang, L. , N. Huang , X. Liu , C. Zhen , and J. Guo . 2023. “The Interaction Effects of Social Support and Four Types of Bullying on Sleep Quality in Chinese Adolescents.” Journal of Affective Disorders 341: 119–127. 37625706 10.1016/j.jad.2023.08.092

[jad12520-bib-0060] Martin‐Storey, A. , R. Legault , and K. C. Prickett . 2020. “Sleep and Its Disorders Among Sexual and Gender Minority Populations.” In Sleep Disorders in Women: A Guide to Practical Management, edited by H. Attarian and M. Viola‐Saltzman , 83–98. Springer International Publishing. 10.1007/978-3-030-40842-8_7.

[jad12520-bib-0061] Martin‐Storey, A. , K. Mayne , W. Beischel , and W. Craig . 2024. “Sleep Health Among Youth Outside of the Gender Binary: Findings From a National Canadian Sample.” Sleep Health 10: 621–627. 10.1016/j.sleh.2024.07.010. 39261146

[jad12520-bib-0062] McKnight‐Eily, L. R. , D. K. Eaton , R. Lowry , J. B. Croft , L. Presley‐Cantrell , and G. S. Perry . 2011. “Relationships Between Hours of Sleep and Health‐Risk Behaviors in US Adolescent Students.” Preventive Medicine 53, no. 4–5: 271–273. 10.1016/j.ypmed.2011.06.020. 21843548

[jad12520-bib-0063] Meanley, S. , D. D. Flores , L. Listerud , C. J. Chang , B. A. Feinstein , and R. J. Watson . 2021. “The Interplay of Familial Warmth and LGBTQ+ Specific Family Rejection on LGBTQ+ Adolescents’ Self‐Esteem.” Journal of Adolescence 93, no. 1: 40–52. 10.1016/j.adolescence.2021.10.002. 34655855

[jad12520-bib-0064] Meldrum, R. C. , D. B. Jackson , R. Archer , and C. Ammons‐Blanfort . 2018. “Perceived School Safety, Perceived Neighborhood Safety, and Insufficient Sleep Among Adolescents.” Sleep Health 4, no. 5: 429–435. 10.1016/j.sleh.2018.07.006. 30241657

[jad12520-bib-0065] Meyer, I. H. 2003. “Prejudice, Social Stress, and Mental Health in Lesbian, Gay, and Bisexual Populations: Conceptual Issues and Research Evidence.” Psychological Bulletin 129, no. 5: 674–697. 10.1037/0033-2909.129.5.674. 12956539 PMC2072932

[jad12520-bib-0066] Meyer, I. H. 2013. “Prejudice, Social Stress, and Mental Health in Lesbian, Gay, and Bisexual Populations: Conceptual Issues and Research Evidence.” Psychology of Sexual Orientation and Gender Diversity 1, no. S: 3–26. 10.1037/2329-0382.1.S.3. PMC2072932 12956539

[jad12520-bib-0067] Miller, K. K. , R. J. Watson , and M. E. Eisenberg . 2020. “The Intersection of Family Acceptance and Religion on the Mental Health of LGBTQ Youth.” Annals of LGBTQ Public and Population Health 1, no. 1: 27–42. 10.1891/LGBTQ.2019-0005.

[jad12520-bib-0068] Morin, A. K. , C. I. Jarvis , and A. M. Lynch . 2007. “Therapeutic Options for Sleep‐Maintenance and Sleep‐Onset Insomnia.” Pharmacotherapy: The Journal of Human Pharmacology and Drug Therapy 27, no. 1: 89–110. 10.1592/phco.27.1.89. 17192164

[jad12520-bib-0069] Morssinkhof, M. W. L. , C. M. Wiepjes , B. W. Bosman , et al. 2023. “Sex Hormones, Insomnia, and Sleep Quality: Subjective Sleep in the First Year of Hormone Use in Transgender Persons.” Sleep Medicine 107: 316–326. 10.1016/j.sleep.2023.04.028. 37271109

[jad12520-bib-0070] Muthén, L. K. , and B. O. Muthén . 2010. Mplus User's Guide: Statistical Analysis With Latent Variables. Muthén & Muthén.

[jad12520-bib-0071] Nagata, J. M. , C. M. Lee , J. H. Yang , et al. 2023. “Sexual Orientation Disparities in Early Adolescent Sleep: Findings From the Adolescent Brain Cognitive Development Study.” LGBT Health 10, no. 5: 355–362. 10.1089/lgbt.2022.0268. 36944127 PMC10316530

[jad12520-bib-0072] O'Leary, K. , L. M. Bylsma , and J. Rottenberg . 2017. “Why Might Poor Sleep Quality Lead to Depression? A Role for Emotion Regulation.” Cognition and Emotion 31, no. 8: 1698–1706. 10.1080/02699931.2016.1247035. 27807996 PMC6190702

[jad12520-bib-0073] Olson, K. R. , L. Durwood , M. DeMeules , and K. A. McLaughlin . 2016. “Mental Health of Transgender Children Who Are Supported in Their Identities.” Pediatrics 137, no. 3: e20153223. 10.1542/peds.2015-3223. 26921285 PMC4771131

[jad12520-bib-0074] Owens, J. , Adolescent Sleep Working Group, Committee On Adolescence , R. Au , M. Carskadon , R. Millman , et al. 2014. “Insufficient Sleep in Adolescents and Young Adults: An Update on Causes and Consequences.” Pediatrics 134, no. 3: e921–e932. 10.1542/peds.2014-1696. 25157012 PMC8194472

[jad12520-bib-0075] Parker, K. , J. Menasce Horowitz , and A. Brown . 2022. Americans’ Complex Views on Gender Identity and Transgender Issues. Pew Research Center. https://www.pewresearch.org/social-trends/2022/06/28/americans-complex-views-on-gender-identity-and-transgender-issues/.

[jad12520-bib-0076] Paruthi, S. , L. J. Brooks , C. D'Ambrosio , et al. 2016. “Consensus Statement of the American Academy of Sleep Medicine on the Recommended Amount of Sleep for Healthy Children: Methodology and Discussion.” Journal of Clinical Sleep Medicine 12, no. 11: 1549–1561. 10.5664/jcsm.6288. 27707447 PMC5078711

[jad12520-bib-0077] Pascoe, C. J. 2005. “Dude, You'Re a Fag’: Adolescent Masculinity and the Fag Discourse.” Sexualities 8, no. 3: 329–346. 10.1177/1363460705053337.

[jad12520-bib-0078] Poteat, V. P. , J. N. Fish , and R. J. Watson . 2021. “Gender‐Sexuality Alliances as a Moderator of the Association Between Victimization, Depressive Symptoms, and Drinking Behavior Among LGBTQ+ Youth.” Drug and Alcohol Dependence 229, no. Part B: 109140. 10.1016/j.drugalcdep.2021.109140. 34775154 PMC8665138

[jad12520-bib-0079] Price‐Feeney, M. , A. E. Green , and S. H. Dorison . 2021. “Impact of Bathroom Discrimination on Mental Health Among Transgender and Nonbinary Youth.” Journal of Adolescent Health 68, no. 6: 1142–1147. 10.1016/j.jadohealth.2020.11.001. 33288457

[jad12520-bib-0080] Reisner, S. L. , K. Conron , N. Scout , M. J. Mimiaga , S. Haneuse , and S. B. Austin . 2014. “Comparing In‐Person and Online Survey Respondents in the U.S. National Transgender Discrimination Survey: Implications for Transgender Health Research.” LGBT Health 1, no. 2: 98–106. 10.1089/lgbt.2013.0018. 26789619

[jad12520-bib-1081] Renley, B. M. , E. Burson , K. A. Simon , A. E. Caba , and R. J. Watson . 2022. “Youth‐Specific Sexual and Gender Minority State‐Level Policies: Implications for Pronoun, Name, and Bathroom/Locker Room use Among Gender Minority Youth.” Journal of Youth and Adolescence 51, no. 4: 780–791. 10.1007/s10964-022-01582-9. 35171396

[jad12520-bib-0081] Robinson‐Cimpian, J. P. 2014. “Inaccurate Estimation of Disparities Due to Mischievous Responders: Several Suggestions to Assess Conclusions.” Educational Researcher 43, no. 4: 171–185. 10.3102/0013189X14534297.

[jad12520-bib-0082] Russon, J. , L. Smithee , S. Simpson , S. Levy , and G. Diamond . 2022. “Demonstrating Attachment‐Based Family Therapy for Transgender and Gender Diverse Youth With Suicidal Thoughts and Behavior: A Case Study.” Family Process 61, no. 1: 230–245. 10.1111/famp.12677. 34046893

[jad12520-bib-0083] Silvers, J. A. 2022. “Adolescence as a Pivotal Period for Emotion Regulation Development.” Current Opinion in Psychology 44: 258–263. 10.1016/j.copsyc.2021.09.023. 34781238

[jad12520-bib-1084] Simon, K. A. , A. E. Caba , B. M. Renley , L. A. Eaton , and R. J. Watson . 2024. “Evidence of Differences in Gender‐Affirming School Experiences in a Sample of Transgender and Gender‐Diverse Youth.” Annals of LGBTQ Public and Population Health 5, no. 3: 176–190. 10.1891/LGBTQ-2023-0006. PMC12629635 41268479

[jad12520-bib-0084] Snyder, E. , B. Cai , C. DeMuro , M. F. Morrison , and W. Ball . 2018. “A New Single‐Item Sleep Quality Scale: Results of Psychometric Evaluation in Patients With Chronic Primary Insomnia and Depression.” Journal of Clinical Sleep Medicine 14, no. 11: 1849–1857. 10.5664/jcsm.7478. 30373688 PMC6223557

[jad12520-bib-0085] Sutherland, D. K. 2019. “The Push for Transgender Inclusion: Exploring Boundary Spanning in the Gay–Straight Alliance.” Sociology Compass 13, no. 11: e12739. 10.1111/soc4.12739.

[jad12520-bib-0086] Tavernier, R. , and T. Willoughby . 2014. “Bidirectional Associations Between Sleep (Quality and Duration) and Psychosocial Functioning Across the University Years.” Developmental Psychology 50, no. 3: 674–682. 23978302 10.1037/a0034258

[jad12520-bib-0087] Tomova, L. , J. L. Andrews , and S.‐J. Blakemore . 2021. “The Importance of Belonging and the Avoidance of Social Risk Taking in Adolescence.” Developmental Review 61: 100981. 10.1016/j.dr.2021.100981.

[jad12520-bib-0088] Toomey, R. B. , C. Ryan , R. M. Diaz , and S. T. Russell . 2011. “High School Gay–Straight Alliances (GSAs) and Young Adult Well‐Being: An Examination of GSA Presence, Participation, and Perceived Effectiveness.” Applied developmental science 15, no. 4: 175–185. 10.1080/10888691.2011.607378. 22102782 PMC3217265

[jad12520-bib-0089] United States Census Bureau . (2022, February 24). Census Bureau Releases New Educational Attainment Data, United States Department of Commerce. https://www.census.gov/newsroom/press-releases/2022/educational-attainment.html.

[jad12520-bib-0003] van Anders, S. M. 2015. “Beyond Sexual Orientation: Integrating Gender/Sex and Diverse Sexualities via Sexual Configurations Theory.” Archives of Sexual Behavior 44, no. 5: 1177–1213. 10.1007/s10508-015-0490-8. 25772652

[jad12520-bib-0004] van Anders, S. M. , and E. J. Dunn . 2009. “Are Gonadal Steroids Linked With Orgasm Perceptions and Sexual Assertiveness in Women and Men?” Hormones and Behavior 56, no. 2: 206–213. 10.1016/j.yhbeh.2009.04.007. 19409392

[jad12520-bib-0090] Watson, R. J. , J. N. Fish , V. P. Poteat , et al. 2021. “Teacher Support, Victimization, and Alcohol Use Among Sexual and Gender Minority Youth: Considering Ethnoracial Identity.” Prevention Science 22, no. 5: 590–601. 10.1007/s11121-021-01216-9. 33609259 PMC8195836

[jad12520-bib-0091] Watson, R. J. , J. F. Veale , and E. M. Saewyc . 2017. “Disordered Eating Behaviors Among Transgender Youth: Probability Profiles From Risk and Protective Factors.” International Journal of Eating Disorders 50, no. 5: 515–522. 10.1002/eat.22627. 27862124 PMC5754211

[jad12520-bib-0092] Wong, M. L. , J. M. Nagata , and M. Barreto . 2024. “Sleep and Socioemotional Outcomes Among Sexual and Gender Minority Adolescents: A Longitudinal Study.” Archives of Sexual Behavior 53, no. 2: 543–553. 10.1007/s10508-023-02732-1. 37993697 PMC11078824

